# Exosome-Mediated miRNA Delivery Restores Early Differentiation and Survival Programs in DGCR8-Deficient Mouse Embryonic Stem Cells

**DOI:** 10.3390/ijms27073000

**Published:** 2026-03-25

**Authors:** Tae-Won Ha, Hyun Kyu Kim, Dongyue No, Jeong Bin Lee, Ahyeon Kim, Bomi Kim, Yena Song, Munkhzul Choijamts, Youngsok Choi, Mihye Lee, Man Ryul Lee

**Affiliations:** 1 Soonchunhyang Institute of Medi-bio Science (SIMS), Soon Chun Hyang University, Cheonan 31151, Republic of Korea; tae-won.ha@weizmann.ac.il (T.-W.H.); hnkkim@health.ucdavis.edu (H.K.K.); mihyelee@sch.ac.kr (M.L.); 2 Department of Molecular Genetics, Weizmann Institute of Science, Rehovot 7610001, Israel; 3 Department of Neurology, School of Medicine, UC Davis, 4625 2nd Ave., Room #3101, Sacramento, CA 95817, USA; 4 Department of Stem Cell and Regenerative Biotechnology, KU Institute of Science and Technology, The Institute of Advanced Regenerative Science, Konkuk University, 120 Neungdong-ro, Gwangjin-gu, Seoul 05029, Republic of Koreachoiys911@gmail.com (Y.C.)

**Keywords:** mouse embryonic stem cells, DiGeorge syndrome critical region 8 (Dgcr8), exosome, microRNA (miRNA), morphogenetic apoptosis

## Abstract

Pluripotent stem cell (PSC) differentiation is orchestrated by intricate autocrine and paracrine signaling networks. Among these, exosomes, key components of the cellular secretome, are implicated as crucial mediators of intercellular communication via delivery of bioactive molecules, including microRNAs (miRNAs). This study investigated the role of exosomal miRNAs in stem cell differentiation using *Dgcr8*-deficient mouse embryonic stem cells (mESCs), which are incapable of producing mature miRNAs. Although the differentiation capacity was markedly impaired in these cells, partial restoration was observed following treatment with exosomes derived from differentiating wild-type mESCs. Exosomal miRNA uptake was confirmed, and gene ontology analysis revealed significant enrichment of pathways associated with cell fate determination, morphogenesis, and apoptosis regulation. Kyoto Encyclopedia of Genes and Genomes pathway analysis indicated that exosomal miRNAs modulated multiple osteoinductive signaling cascades, notably the MAPK and TGF-β pathways, in Dgcr8-deficient cells. Apoptotic markers were also downregulated, suggesting a protective effect conferred by the exosomal cargo. Collectively, our results suggest that exosome-mediated delivery of miRNAs may represent a fundamental mechanism by which pluripotent stem cells coordinate stress responses and differentiation trajectories, providing novel insights into the regulation of embryogenesis.

## 1. Introduction

Understanding early embryonic development and cell fate specification by studying embryonic stem cells (ESCs) is essential from both scientific and medical perspectives [1,2]. During early development, morphogens, signaling molecules that regulate patterning and organogenesis, play pivotal roles in determining spatial organization and lineage specification of specialized cell types [3,4,5].

These morphogens regulate target cell behavior by activating signaling pathways that control proliferation, differentiation and pattern formation.

Recently, exosomes, a class of extracellular vesicles enclosed by lipid bilayers, have been identified as novel mediators of intercellular communication under both physiological and pathological conditions [6]. These vesicles, typically ranging from 50 to 150 nm in diameter, are generated via the inward budding of multivesicular bodies (MVBs) and are released into the extracellular space upon the fusion of MVBs with the plasma membrane [7,8]. Exosomes have been isolated from a wide range of biological fluids and cell culture supernatants, and their cargo, comprising proteins, lipids, and nucleic acids, exerts diverse biological effects [9,10,11,12]. Exosome-mediated delivery reportedly contributes to key developmental signaling processes, including Wnt, Hedgehog, Notch, and BMP pathways [13,14,15,16]. In addition, exosomes secreted by mesenchymal stem cells, immune cells, and tumor cells have been implicated in various biological processes, including organogenesis, immune modulation, tumor progression, metastasis, and drug resistance [17]. The molecular content of exosomes varies depending on the cell type and physiological state and is tightly regulated by context-dependent mechanisms [18].

Among the molecular components of exosomes, diverse classes of messenger RNAs (mRNAs) and microRNAs (miRNAs) have been identified through microarray and next-generation sequencing analyses [19,20,21]. Notably, some specific RNAs are more abundant in exosomes than in their donor cells, suggesting the existence of selective RNA packaging mechanisms [22]. These RNA cargos can be transported over long distances via circulating exosomes and delivered to recipient cells, where they retain the capacity to modulate gene expression [23]. Exosomal mRNAs can be translated into functional proteins, while miRNAs can post-transcriptionally repress target gene expression, thereby extending their functional roles beyond intracellular regulation [24]. Given their capacity to carry regulatory RNA species, exosomes are increasingly recognized as important modulators of early embryonic development. Traditionally, processes, such as programmed cell death, cellular migration, lineage commitment, and tissue patterning, are considered fundamental to early embryogenesis. It has been hypothesized that miRNAs contained in exosomes secreted by early embryonic cells may serve as a new class of morphogens capable of fine-tuning developmental trajectories. To explore this possibility, we employed a *Dgcr8*-knockout ESC model in which the biogenesis of mature miRNAs is severely disrupted due to the loss of Drosha-*Dgcr8* complex activity [25]. In the canonical miRNA biogenesis pathway, primary miRNAs (pri-miRNAs) are transcribed by RNA polymerase II and processed into hairpin-shaped precursor miRNAs (pre-miRNAs) by the nuclear RNase III enzyme, Drosha. These approximately 70-nucleotide pre-miRNAs are then exported to the cytoplasm by Exportin-5 and further cleaved by cytoplasmic RNase III (Dicer) to produce mature miRNAs [26]. The Drosha complex, consisting of Drosha and *Dgcr8*, plays a crucial role in the recognition and processing of pri-miRNAs [27,28]. Although *Dgcr8*-deficient ESCs exhibit normal levels of pri-miRNA transcription, they show a dramatic reduction in mature miRNA production due to defective Drosha complex function.

Given this unique feature, *Dgcr8*-deficient ESCs provide a powerful model system for dissecting the role of mature miRNAs in early development [25]. This study aimed to elucidate the mechanism by which exosome-mediated delivery of miRNAs contributes to intercellular communication during early embryogenesis, with the aim of identifying novel miRNA-dependent regulatory mechanism. Toward this, wild-type and *Dgcr8*-knockout ESCs were subjected to differentiation, and the exosomes secreted during this process were isolated for miRNA profiling.

## 2. Results

### 2.1. Dgcr8 Is Essential for miRNA Biogenesis but Non-Essential for the Undifferentiated Stem Cell State

**Figure 1:**
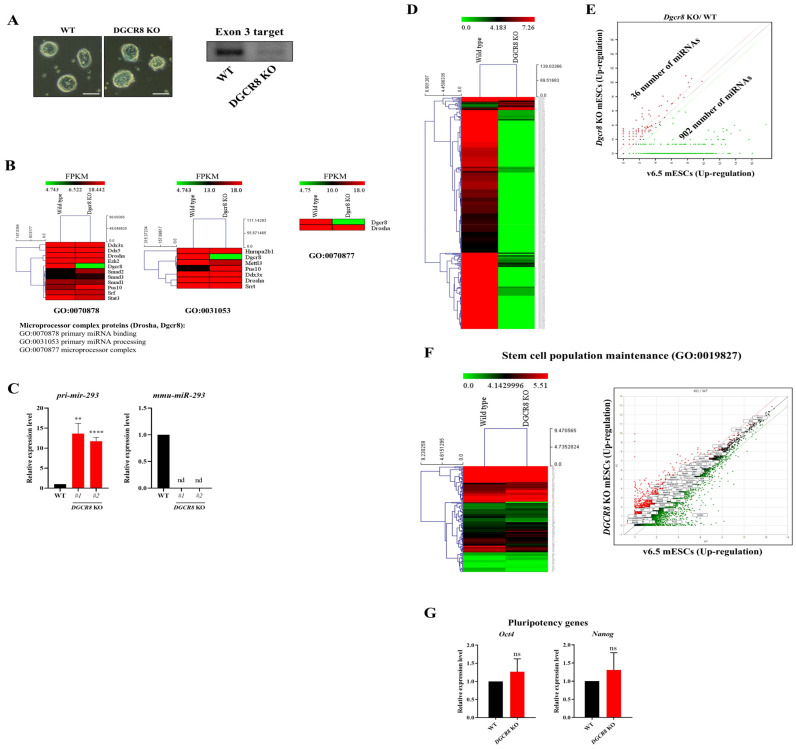
Characterization of *Dgcr8* knockout (KO) mouse embryonic stem cells (mESCs) and transcriptomic profiling of miRNA biogenesis and stemness-related genes. (**A**) Representative bright-field images of wild-type (WT) and *Dgcr8* KO mESC colonies cultured under standard conditions (scale bars = 100 μm), and immunoblot analysis showing the absence of Dgcr8 protein in KO cells. (**B**) Heatmap of RNA-seq data showing the expression of genes associated with the microprocessor complex (GO:0070877), primary miRNA binding (GO:0070878), and primary miRNA processing (GO:0031053). Among these, only *Dgcr8* was significantly downregulated in *Dgcr8* KO mESCs, validating the specificity of the KO. (**C**) RT-PCR analysis of pri-miR-293 and mature miR-293 expression in WT and *Dgcr8* KO mESCs. Data are presented as mean ± SD. Tukey’s post hoc test: ** *p* < 0.01; **** *p* < 0.0001. nd, not detected. (**D**) Heatmap displaying global miRNA expression profiles in WT and *Dgcr8* KO mESCs. (**E**) Scatter plot comparing miRNA expression levels between WT and *Dgcr8* KO mESCs. Most of the miRNAs fell below the two-fold downregulation threshold; a substantial number of miRNAs (902) were downregulated, while a small subset (36) were upregulated in KO cells. (**F**) Despite the widespread reduction in miRNA expression, transcriptomic analysis revealed that genes involved in the maintenance of the stem cell population (GO:0019827) were largely unaffected in *Dgcr8* KO cells, as shown in the heatmap and scatter plot. (**G**) RT-qPCR analysis of core pluripotency factors Oct4 and Nanog in WT and *Dgcr8* KO mESCs. No significant differences were observed, indicating that canonical miRNA loss did not impair the maintenance of pluripotency at the transcript level (ns = not significant; mean ± SD).

During early embryogenesis, intercellular communication via secreted factors is essential for guiding cell-fate decisions [29]. Among these, exosomal miRNAs have emerged as key regulators of lineage specification and stem cell differentiation [30,31]. To determine the functional contribution of exosome-mediated miRNA transfer during early differentiation, we employed Dgcr8 knockout (KO) mouse embryonic stem cells (mESCs) in which exon 3 of the *Dgcr8* gene encoding the RNA-binding domain, is deleted [32]. This model system was used to analyze miRNA biogenesis and exosomal miRNA communication in a Dgcr8-deficient background. To confirm the genotype of the obtained cell line, we performed PCR using genomic DNA templates, which verified the deletion of exon 3 (Figure 1A, right panel). Morphologically, Dgcr8 KO mESCs formed compact colonies similar to those of wild-type (WT) cells, suggesting retention of the undifferentiated state under standard culture conditions (Figure 1A, left panel). To assess molecular changes associated with *Dgcr8* deletion, RNA sequencing was performed on WT and KO mESCs. Gene expression profiles related to the microprocessor complex, specifically primary miRNA binding (Gene Ontology (GO):0070878), primary miRNA processing (GO:0031053), and microprocessor complex (GO:0070877), were compared. Heatmap analysis of FPKM values revealed that only Dgcr8 expression was abolished in KO cells, while other components, including Drosha, Ddx5, and Ddx17, remained unchanged (Figure 1B), consistent with the selective KO of *Dgcr8* [33]. To verify the functional consequence of *Dgcr8* loss on canonical miRNA processing, we analyzed the expression of pri-miR-293 and mature miR-293, a well-characterized miRNA enriched in mESCs [34]. RT-qPCR analysis revealed significant accumulation of pri-miR-293 in KO cells (*p* < 0.01 and *p* < 0.0001), whereas mature miR-293 was undetectable (Figure 1C). These results indicated that the loss of *Dgcr8* blocked pri-miRNA processing, leading to nuclear retention of unprocessed transcripts.

To examine the global impact of *Dgcr8* depletion on miRNA expression, we performed RNA-seq in WT and *Dgcr8* KO mESCs. A total of 1976 miRNAs were detected in both groups. Of these, 902 miRNAs were significantly downregulated in KO cells, while only 36 were upregulated (Figure 1D,E). The heat map in Figure 1D clearly illustrates the widespread reduction in miRNA expression in Dgcr8-deficient cells, consistent with the loss of canonical miRNA processing activity. Scatter plot comparison (Figure 1E) further highlighted the asymmetric distribution of changes, with the majority of downregulated miRNAs clustered below the diagonal line. These results confirm that Dgcr8 is essential for the biogenesis of several miRNAs in mESCs.

Despite the global reduction in mature miRNA expression, the transcriptomic profiles of genes involved in stem cell population maintenance (GO:0019827) remained largely unchanged between *Dgcr8* KO and WT mESCs. RNA-seq revealed no significant differences in the expression of key genes within this GO term, as shown in the heatmap and scatter plot (Figure 1F). Furthermore, RT-qPCR demonstrated that the expression levels of the core pluripotency markers Oct4 and Nanog were not significantly different between KO and WT cells (Figure 1G). These findings indicate that the undifferentiated state and transcriptional identity of mESCs were maintained in the absence of canonical miRNA processing, at least under standard self-renewal culture conditions.

### 2.2. Dgcr8 Is Essential for the Induction of Lineage-Specific Markers During mESC Differentiation

**Figure 2:**
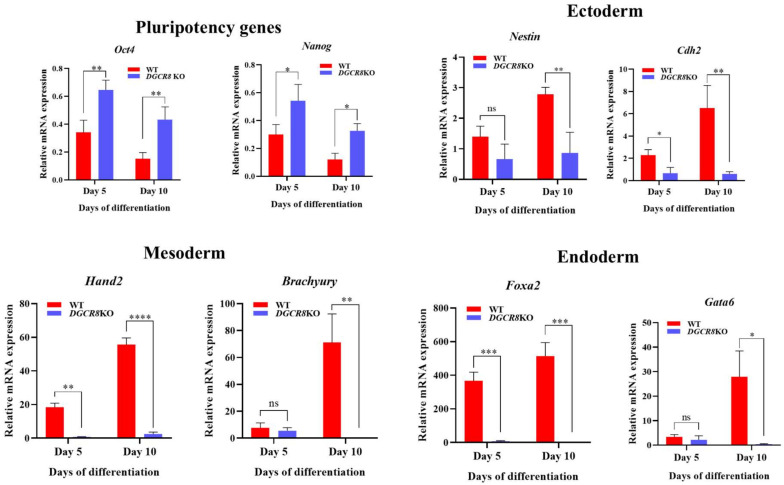
Loss of *Dgcr8* disrupts lineage-specific gene induction during spontaneous differentiation of mouse embryonic stem cells (mESCs). Embryoid bodies (EBs) were generated from WT and *Dgcr8* knockout (KO) mESCs and cultured without leukemia inhibitory factor (LIF) to induce spontaneous differentiation. Gene expression levels of pluripotency markers (*Oct4*, *Nanog*), epithelial/mesenchymal markers (*Cdh1*, *Cdh2*), and lineage-specific markers, including those of the ectoderm (Nestin), primitive streak, and mesendoderm (*Brachyury*, *Foxa2*, *Mixl1*), mesoderm (*Hand1*, *Hand2*, *Twist1*), and endoderm (*Gata4*, *Gata6*), were evaluated at day 5 and day 10 using RT-qPCR. All values were normalized to *Gapdh* and are shown relative to those of undifferentiated WT ESCs as baseline control. Values represent the mean ± SD (*n* = 3). Tukey’s post hoc test: * *p* < 0.05; ** *p* < 0.01; *** *p* < 0.001; **** *p* < 0.0001; ns = not significant.

To determine whether the absence of mature miRNAs in Dgcr8-deficient mESCs impairs their ability to differentiate, we assessed the expression of pluripotency and lineage-specific markers during embryoid body (EB) formation. mESCs were cultured in suspension without leukemia inhibitory factor (LIF) for 5 or 10 days to allow spontaneous differentiation, and gene expression was analyzed by RT-qPCR. As expected, WT EBs showed progressive downregulation of *Oct4* and *Nanog*, key transcription factors maintaining pluripotency, during differentiation. In contrast, *Dgcr8* KO EBs exhibited delayed reduction in *Oct4* and *Nanog* expression, with both genes remaining significantly elevated on days 5 and 10 compared to levels in WT EBs (Figure 2, top left). These data suggest that Dgcr8-deficient cells partially retain their pluripotent characteristics under differentiation-inducing conditions. Next, we evaluated the expression of representative markers of the three germ layers [25,35]. In WT EBs, the expression of the ectodermal markers, *Nestin* and *Cdh2*, was robustly induced over the course of differentiation. However, *Dgcr8* KO EBs showed significantly reduced induction of both genes on days 5 and 10 (Figure 2, top right).

Similarly, the mesodermal markers *Hand2* and *Brachyury* (T) were strongly upregulated in WT cells over time, whereas *Dgcr8* KO cells showed minimal induction, particularly in Hand2 cells, wherein expression remained nearly undetectable even after 10 days (Figure 2, bottom left). The expression of the endodermal markers *Foxa2* and *Gata6* followed the same trend, with robust upregulation in WT EBs and markedly attenuated expression in Dgcr8 KO EBs, indicating a global impairment in endodermal lineage commitment (Figure 2, bottom right). These results support a role for Dgcr8-dependent miRNAs in the coordination of early differentiation across all germ layers.

### 2.3. Co-Culture with WT Cells Restores Germ Layer Marker Expression in Dgcr8-Deficient mESCs

**Figure 3:**
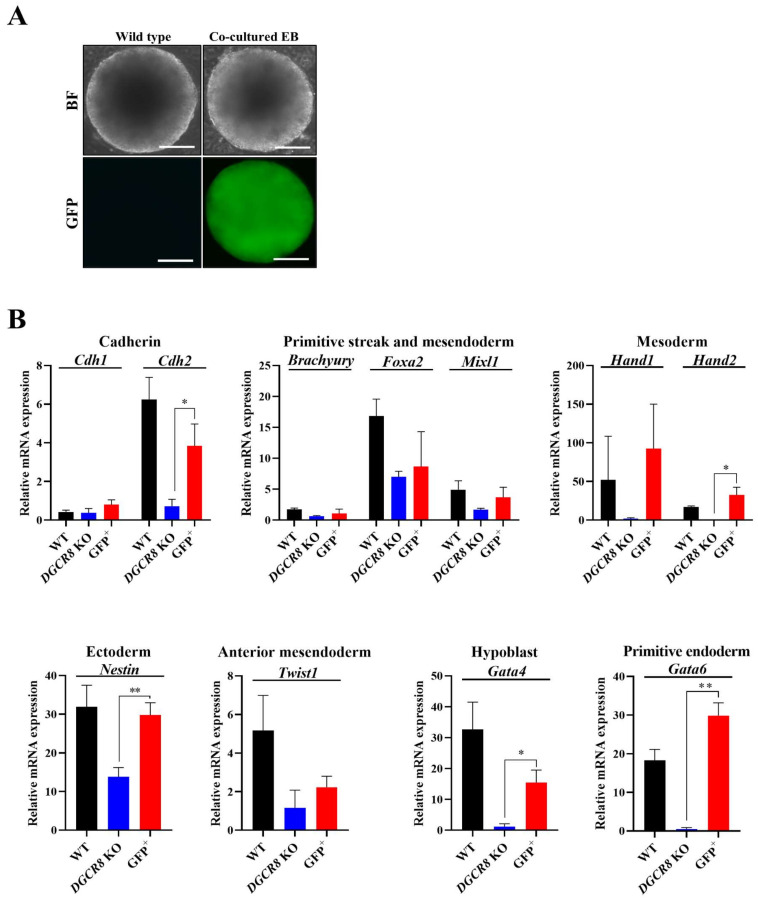
Co-culture with wild-type mouse embryonic stem cells (mESCs) partially restores differentiation capacity in *Dgcr8* knockout (KO) cells. To assess whether extrinsic cues from wild-type ESCs can rescue the differentiation defects of *Dgcr8*-deficient ESCs, embryoid bodies (EBs) were generated by co-culturing green fluorescent protein (GFP)-labeled *Dgcr8* KO and wild-type ESCs at a 1:1 ratio. (**A**) Bright field (top) and GFP fluorescence (bottom) images show the morphology of mixed EBs at day 10, confirming proper aggregation of both cell types. Scale bar = 100 μm. (**B**) To examine lineage-specific gene expression in *Dgcr8* KO cells, GFP+ cells were isolated from the mixed EBs via FACS on day 10. RT-qPCR analysis of sorted GFP+ KO cells revealed significant restoration of *Nestin*, *Hand2*, and *Gata6* expression. Partial rescue was observed for *Cdh2* and *Gata4*. Foxa2, *Mixl1*, and *Twist1* showed upward trends that were not statistically significant. Values represent the mean ± SD (*n* = 3). Statistical analyses was performed using Tukey’s post hoc test. * *p* < 0.05; ** *p* < 0.01.

To determine whether the intercellular transfer of miRNAs influences differentiation, we established a co-culture system by mixing *Dgcr8*-KO mESCs, which lack canonical miRNA biogenesis, with WT mESCs at a 1:1 ratio. To distinguish Dgcr8 KO cells, they were stably transduced to express green fluorescent protein (GFP) prior to EB formation. During differentiation, GFP expression was maintained, enabling tracking of KO-derived cells in the co-culture (Figure 3A). The mixed population was cultured under LIF-free differentiation conditions for 10 days, after which GFP-positive cells were isolated using fluorescence-activated cell sorting (FACS). Thereafter, RT-qPCR was performed to evaluate the expression of lineage-specific markers in sorted KO cells that had been co-cultured with WT cells. Compared with *Dgcr8* KO cells differentiated alone, co-cultured KO cells showed a significant increase in germ layer marker expression. Notably, *Nestin* (ectoderm, *p* < 0.01), *Hand2* (mesoderm, *p* < 0.05), and *Gata6* (endoderm, *p* < 0.01) expression was restored to near WT levels. Additional recovery was observed for *Cdh2* (*p* < 0.05) and *Gata4* (*p* < 0.05), while markers, such as *Foxa2*, *Mixl1*, and *Twist1*, showed upward trends without statistical significance. These results indicate that cell-to-cell communication during early differentiation enables the transfer of miRNA-dependent regulatory signals, which can compensate for intrinsic deficiencies in miRNA biogenesis.

### 2.4. Exosomal miRNAs from WT EBs Restore Lineage Gene Expression in Dgcr8-Deficient mESCs

**Figure 4:**
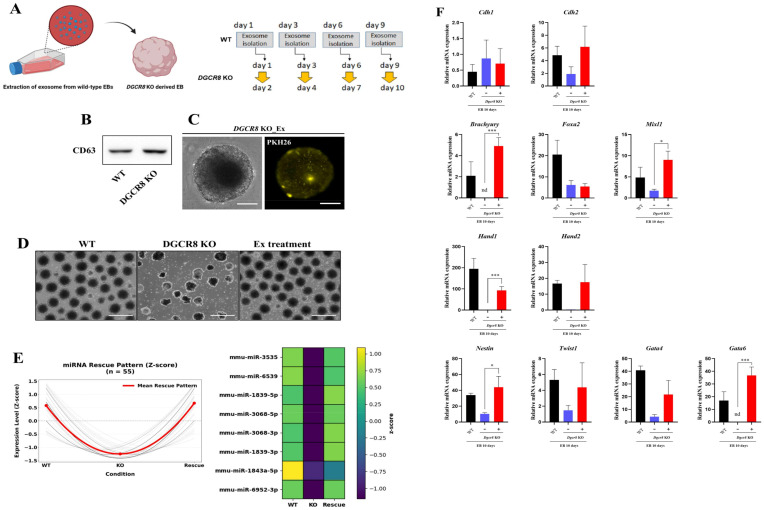
Exosomes derived from differentiating wild-type (WT) embryonic stem cells (ESCs) restore lineage gene expression in *Dgcr8*-deficient ESCs. (**A**) Schematic diagram of the experimental workflow. Exosomes were isolated from differentiating WT embryoid bodies (EBs) every 2–3 days (days 1, 3, 6, and 9) and sequentially injected into *Dgcr8* knockout (KO) EBs until day 10 of differentiation. (**B**) Western blot analysis showing CD63 expression in both WT and *Dgcr8* KO EBs. (**C**) Uptake of PKH26-labeled WT-derived exosomes into *Dgcr8* KO EBs was visualized using fluorescence microscopy. Scale bar = 100 μm. (**D**) Bright field images of day 10 EBs from WT, *Dgcr8* KO, and exosome-treated *Dgcr8* KO ESCs. Scale bar = 1000 μm. (**E**) miRNA-seq of WT, Dgcr8 KO, and exosome-treated KO (Rescue) EBs identified 55 miRNAs with a shared rescue trajectory (WT-high, KO-low, rescue-recovered). Left: Z-score–normalized expression traces (gray) with mean pattern (red). Right: heatmap of representative rescued miRNAs (color scale, Z-score). (**F**) RT-qPCR analysis of lineage-specific gene expression at day 10. Exosome-treated *Dgcr8* KO EBs showed increased expression of *Nestin*, *Cdh2* (ectoderm); *Brachyury*, *Hand2*, *Mixl1* (mesoderm); and *Gata6*, *Foxa2*, *Gata4*, *Twist1* (endoderm). Data represent mean ± SD (*n* = 3). nd, not detected. Statistical analysis was performed using Tukey’s post hoc test: * *p* < 0.05; *** *p* < 0.001.

Given the observed recovery of differentiation markers in *Dgcr8* KO cells co-cultured with WT ESCs (Figure 3), we investigated whether this effect could be mediated by exosome-derived miRNAs. Exosomes were isolated every two days from differentiating WT EBs and administered to *Dgcr8* KO EBs at 40 μg/mL throughout a 10-day differentiation period (Figure 4A). Immunoblot analysis confirmed the expression of the exosomal marker CD63 in both WT and KO cells (Figure 4B). The uptake of PKH26-labeled exosomes into KO EBs was visualized by fluorescence microscopy (Figure 4C). Morphologically, exosome-treated KO EBs exhibited increased compactness and a spherical shape compared to untreated KO EBs, resembling WT EBs (Figure 4D). To identify the exosome-responsive miRNA cargo associated with phenotypic rescue, we performed miRNA sequencing of WT EBs (day 7), untreated *Dgcr8* KO EBs, and exosome-treated KO EBs. Overall, the majority of miRNAs were markedly downregulated under the KO condition, consistent with the loss of mature miRNAs caused by *Dgcr8* deficiency, whereas exosome treatment restored a subset of miRNAs to WT-like levels. Using pattern-based analysis with Z-score–normalized expression values, we extracted a subset of 55 miRNAs displaying a characteristic rescue trajectory: high in WT, reduced in KO, and re-elevated upon exosome treatment (Figure 4E). The aggregated behavior of these miRNAs formed a consistent V-shaped recovery profile, indicating coordinated reconstitution of the miRNA landscape under rescue conditions. Representative miRNAs with robust recovery dynamics were further visualized as a heatmap, which confirmed that KO-associated decreases and rescue-associated reconstitutions were consistently observed at the level of individual miRNAs (Figure 4E). RT-qPCR analysis on day 10 revealed restored expression of germ layer-specific genes in exosome-treated KO cells (Figure 4F). Notably, *Cdh2* and *Nestin* (ectoderm) were upregulated to WT levels. *Brachyury* (*p* < 0.001), *Mixl1* (*p* < 0.05), and *Hand2* (*p* < 0.0001) were significantly recovered. *Gata6*, which was not detected in untreated KO EBs, was induced by exosome treatment (*p* < 0.01). Additional recovery was observed for *Foxa2*, *Twist1*, and *Gata4*. These results demonstrate that exosomes derived from differentiating ESCs deliver functional miRNAs that can restore differentiation defects in *Dgcr8* KO mESCs, supporting a model in which miRNA-containing exosomes act as morphogen-like signals during early development [36].

### 2.5. Exosome-Mediated Rescue Is Associated with Reactivation of Apoptosis-, Chromatin-, and Morphogenesis-Related Transcriptional Programs

**Figure 5:**
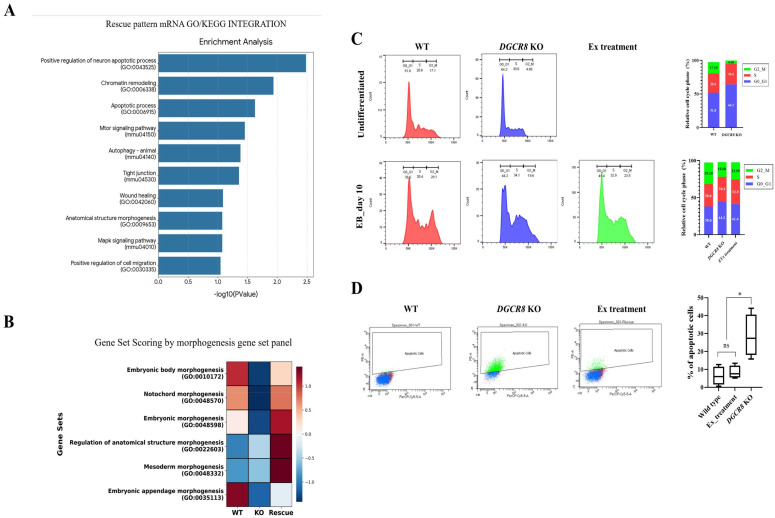
Exosome treatment modulates transcriptional programs, cell cycle, and apoptosis in *Dgcr8*-deficient embryoid bodies (EBs). (**A**) Integrated Gene Ontology (GO)/Kyoto Encyclopedia of Genes and Genomes (KEGG) enrichment analysis of rescue-pattern mRNA candidates identifies significant enrichment of apoptosis-, chromatin remodeling-, and stress signaling-related pathways. Bar length indicates −log10(*p* value). (**B**) Gene set scoring heatmap for morphogenesis-related GO gene sets across WT, *Dgcr8* knockout (KO), and rescue conditions, showing suppression in KO and recovery upon rescue. Color scale indicates relative gene-set score (higher, red; lower, blue). (**C**) DNA-content flow cytometry profiles and quantified phase distribution for WT and *Dgcr8* KO cells in the undifferentiated state (top) and for day 10 EBs from WT, *Dgcr8* KO, and exosome-treated KO conditions (bottom). Percentages of cells in the G0/G1, S, and G2/M phases are indicated. (**D**) Flow cytometry-based apoptosis analysis of day 10 EBs. Representative plots (left) and quantification of apoptotic cells (right) show increased apoptosis in *Dgcr8* KO and reduction following exosome treatment. ns, not significant; * *p* < 0.05.

To elucidate the downstream molecular programs potentially regulated by the rescued miRNA pool, we prioritized mRNA candidates putatively targeted by the rescue-pattern miRNAs identified in WT, *Dgcr8* KO, and exosome-treated (rescue) EBs and subjected these transcripts to integrated GO and Kyoto Encyclopedia of Genes and Genomes (KEGG) pathway enrichment analyses. These analyses revealed a strong enrichment of gene sets related to apoptosis and cell survival control, with positive regulation of neuron apoptotic process (GO:0043525) emerging as the most statistically significant term, followed by chromatin remodeling (GO:0006338) and the broader apoptotic process (GO:0006915) category (Figure 5A). In addition, multiple pathways implicated in stress adaptation and differentiation-associated signaling were significantly enriched, including mTOR signaling (mmu04150), MAPK signaling (mmu04010), and autophagy (mmu04140), together with processes related to tissue organization and remodeling, such as tight junction (mmu04530), wound healing (GO:0042060), anatomical structure morphogenesis (GO:0009653), and positive regulation of cell migration (GO:0030335) (Figure 5A). Collectively, these results indicate that the transcriptomic restoration observed under rescue conditions is not limited to lineage-marker recovery but is also accompanied by coordinated rebalancing of cell death checkpoints, epigenetic/chromatin regulatory programs, and major kinase-driven signaling cascades that support cellular homeostasis during early differentiation.

To more specifically evaluate the impact of rescue on developmental architecture, we performed gene set scoring using a curated morphogenesis-related gene set panel and visualized the scores across WT, KO, and rescue conditions. Consistent with the morphological defects observed in *Dgcr8* KO EBs, morphogenesis-associated gene sets, including embryonic body morphogenesis (GO:0010172) and notochord morphogenesis (GO:0048570), were broadly suppressed in the KO group (Figure 5B). Notably, the rescue condition showed robust reactivation of several key programs, including embryonic morphogenesis (GO:0048598), regulation of anatomical structure morphogenesis (GO:0022603), and mesoderm morphogenesis (GO:0048332), reaching or exceeding the WT level scores in some cases (Figure 5B). These data support the interpretation that exosome-mediated rescue reinstates transcriptional networks governing morphogenetic progression and tissue patterning programs, providing a mechanistic link between miRNA restoration and improved EB organization during early differentiation.

To functionally validate the rescue-associated pathways suggested by integrative transcriptomic analyses, we assessed cell-cycle distribution and apoptosis in WT, *Dgcr8* KO, and exosome-treated EBs (Figure 5C,D). Flow cytometry showed that in the undifferentiated state, *Dgcr8* KO ESCs exhibited a pronounced shift toward G0/G1 with a concomitant reduction in G2/M compared to those of WT cells (G0/G1: 51.8% vs. 64.3%; S: 28.6% vs. 30.6%; G2/M: 17.1% vs. 4.68%, WT vs. KO; Figure 5C, top). Ten days after differentiation, WT EBs displayed a balanced distribution (G0/G1: 38.0%, S: 30.4%, G2/M: 29.1%), whereas *Dgcr8* KO EBs remained skewed toward earlier phases (G0/G1: 44.2%, S: 34.1%), with a reduced G2/M fraction (19.6%) (Figure 5C, bottom). Notably, exosome treatment partially normalized the KO-associated cell cycle distortion, shifting the distribution toward the WT profile (G0/G1: 41.4%; S: 32.9%; G2/M: 23.5%; Figure 5C, bottom). Consistent with the enrichment of apoptosis-related gene sets, flow cytometry-based apoptosis analysis revealed a marked increase in apoptotic cells in *Dgcr8* KO EBs, whereas exosome treatment reduced apoptosis to near WT levels (Figure 5D). Specifically, the apoptotic fraction was substantially higher in the KO group (median, approximately 25–30%, with the upper range reaching approximately 40%) than in the WT group (median, approximately 5–10%). Exosome-treated EBs showed a reduced apoptotic fraction (approximately 8–12%), which was not significantly different from that of WT (ns) but was significantly lower than that of *Dgcr8* KO EBs (Figure 5D). Together, these functional assays corroborated that exosome treatment alleviated cell cycle dysregulation and excess apoptosis associated with *Dgcr8*-dependent miRNA deficiency during EB differentiation, providing mechanistic support for the observed restoration of differentiation-associated phenotypes.

## 3. Discussion

In this study, we introduced a novel conceptual framework in which exosomes function as morphogen-like paracrine mediators of early embryonic differentiation, with exosomal miRNAs playing a central regulatory role.

Exosomes are small extracellular vesicles enriched in bioactive cargo, including miRNAs, mRNAs, proteins, and lipids, and are known to participate in cell-to-cell communication [10,23,37]. Building on these characteristics, we hypothesized, based on our findings, that exosomes may act not merely as passive carriers, but also as active regulators akin to classical morphogens, orchestrating spatial and temporal gene expression programs during early development. To the best of our knowledge, this is the first study to provide experimental evidence supporting the morphogen-like role of exosomal miRNAs in embryonic stem cell-fate specification.

To test this hypothesis, we utilized a *Dgcr8* KO mouse ESC model in which canonical miRNA biogenesis is disrupted [25]. Loss of *Dgcr8*, a core component of the microprocessor complex, resulted in a profound depletion of mature miRNAs and accumulation of unprocessed pri-miRNAs, leading to impaired differentiation across all three germ layers. Interestingly, core pluripotency factors, such as *Oct4* and *Nanog*, remained largely unaffected. While miRNAs are known to contribute to the maintenance of pluripotency, transcription factors, such as *Oct4* and *Nanog*, are thought to play a more central role in sustaining the pluripotent state [38,39,40]. In contrast, the transition from pluripotency to lineage commitment appears to require the coordinated action of both lineage-specifying transcription factors and miRNAs, suggesting a critical role for miRNAs in initiating differentiation. To assess whether extracellular signaling could restore the differentiation potential, *Dgcr8* KO cells were either co-cultured with differentiating WT ESCs during EB formation or treated with exosomes isolated from differentiating WT ESCs. In both cases, significant restoration of lineage-specific gene expression, morphogenetic structure, and cell cycle progression was observed, indicating that intercellular communication, particularly via exosomal miRNAs, can compensate for intrinsic miRNA deficiencies during early differentiation. Given that the only biologically meaningful variables reintroduced were mature miRNAs, we attributed this phenotypic rescue to exosomal miRNAs. This was further substantiated using an miRNA-deficient background, which served as an intrinsic control for isolating the effects of miRNA reintroduction. Although exosomes inherently carry multiple bioactive components [37], the recovery of differentiation in our model, in which the primary defect is miRNA loss, supports the conclusion that exosomal miRNAs are the principal effectors of rescue.

To identify the potential regulatory pathways, we performed miRNA and mRNA sequencing, followed by integrative bioinformatics analyses. Hierarchical clustering revealed six distinct expression clusters, among which subsets of miRNAs and their inversely correlated mRNA targets were enriched in rescued cells but absent in *Dgcr8* KO cells. GO and gene-set enrichment analysis (GSEA) indicated significant involvement of miRNA targets in key processes, including morphogenesis, apoptosis regulation, and cell cycle control. Notably, enriched pathways included the TGF-β and MAPK signaling cascades, both of which are known to regulate early developmental transitions and epithelial–mesenchymal transition/mesenchymal–epithelial transition dynamics [41,42,43].

The most striking observation was the normalization of apoptosis-related phenotypes. Exosomal miRNA treatment restored the expression of genes associated with morphogenetic apoptosis, reduced cell death, and reestablished normal EB morphology. This suggests that modulation of apoptosis via miRNA-mediated paracrine signaling is a critical checkpoint in early differentiation and that exosomes can serve as regulators of this process.

Another intriguing aspect of our findings is that despite the loss of nearly 50% of mature miRNAs in *Dgcr8* KO cells, the restoration of differentiation could be largely achieved by exosomal miRNAs. This implies that a limited subset of miRNAs, rather than the entire miRNA repertoire, is sufficient to trigger key cell-fate transitions. This concept parallels that of cytokine-driven lineage commitment, where broad involvement exists, but specific factors (e.g., FGF in neural induction) are indispensable for initiation [44,45]. Similarly, our results suggest that select exosomal miRNAs serve as core regulators of lineage specification.

However, this study has several limitations. First, although the use of an miRNA-deficient model supported our conclusions, direct functional validation of individual miRNAs, such as gain- or loss-of-function studies using mimics/antagomiRs, was not performed. Future studies should investigate the specific contributions of the candidate miRNAs identified herein. Secondly, although we argue that exosomal miRNAs are the primary mediators of rescue, the possibility that other exosomal components contribute synergistically cannot be excluded. Although we did not perform RNase treatment or use miRNA-depleted vesicles, the *Dgcr8* KO background provides a strong biological filter, minimizing the influence of non-miRNA factors [46,47]. Future studies employing engineered exosomes with selective cargo depletion are necessary to rigorously delineate the roles of proteins, lipids, and mRNAs.

In summary, this study established a novel regulatory role for exosomal miRNAs in restoring early differentiation in an miRNA-deficient stem cell model. Our findings suggest that exosomes may serve as morphogen-like signaling units that transmit critical regulatory cues through miRNA cargo to guide cell fate decisions. This work opens a new avenue for understanding intercellular communication in early development and presents exosomal miRNAs as potential tools for the regenerative modulation of stem cell fate.

## 4. Materials and Methods

### 4.1. Cell Culture

The v6.5 mouse ESC line (Novus Biologicals, Centennial, CO, USA) and *Dgcr8* KO mESC line (Novus Biologicals, CO, USA) were maintained in DMEM-high glucose, supplemented with 15% FBS, 100X non-essential amino acid, 200 mM L-Glutamine (Sigma, St. Louis, MO, USA), 2-mercaptoethanol (Corning, New York, NY, USA), and 10 ng/mL LIF (Sigma, St. Louis, MO, USA) with MEF feeder [48,49]. These cells were cultured on 0.1% gelatin (Wellgene, Gyeongsan, Republic of Korea) in N2B27 medium with LIF and two inhibitors (GSK-3 beta inhibitor, MEK1/2 inhibitor) (DMEM/F12 + 100× N2 supplement, Neurobasal + 50× B27 supplement, CHIR99021, and PD0325901 10 mM both, LIF-107 units/mL) under feeder-free conditions. mESCs were routinely passaged every 3 days using TrypLE Express (Gibco, Grand Island, NY, USA) and passaged every 4–5 days using 0.05% trypsin. All cells were maintained in an incubator at 37 °C with 5% CO_2_.

### 4.2. Induction of EB Differentiation

EB structures were first induced by seeding mESCs into 24-well AggreWellTM plates (STEMCELL Technologies, Vancouver, BC, Canada) [50]. After 1–2 days, the EBs were transferred to a low-attachment 6-well plate containing half the amount of LIF. The plates were incubated on an orbital shaker (New Brunswick Scientific, Enfield, CT, USA) (60 rpm) for a day. After EBs were clearly formed, differentiation was initiated with LIF-free serum medium change (day 1). The medium was changed every 2 days until differentiation day 10.

### 4.3. Total RNA Extraction and Quantitative Real-Time PCR

Cells were lysed with easy-BLUE (iNtRON, Daejeon, Republic of Korea), and total RNA was extracted and quantified using a NanoDrop spectrophotometer (Thermo Fisher Scientific, Waltham, MA, USA). One microgram of RNA was reverse-transcribed using the All-in-One 5× First Strand cDNA Synthesis Master Mix kit (CellScript, Madison, WI, USA), and quantitative PCR was performed using TOPreal™ qPCR 2X PreMIX (Enzynomics, Daejeon, Republic of Korea) according to the manufactures’ protocols. RNA was extracted from each treatment condition, and the experiments were performed in triplicate. Primer sequences were designed according to the Integrated DNA Technologies website (https://sg.idtdna.com/pages, accessed on 12 May 2021) and are shown in Table 1. For miRNA qPCR, the expression levels of miRNAs were determined using TaqMan^TM^ miRNA assay primer, universal PCR master mix, and StepOne sequence detector (Applied Biosystems, Carlsbad, CA, USA). Endogenous *U6* was used as a control miRNA to normalize intracellular miRNA expression. All primers were validated for efficiency, and data were obtained from experiments performed in triplicate.

### 4.4. Total Exosome Isolation

Exosomes were isolated by Total Exosome Isolation Reagent (from cell culture media) (Invitrogen, Carlsbad, CA, USA). To ensure that the exosomes originated from EBs, EBs were cultured for 12 h without FBS. The cell culture medium was harvested and centrifuged at 2000× *g* for 30 min to remove debris and cells. The cell-free culture medium was then transferred to a new tube, followed by addition of 0.5 volume of the Total Exosome Isolation (from cell culture medium) reagent, mixing via vortexing to obtain a homogenous solution, and incubation at 4 °C overnight. Thereafter, the sample was centrifuged at 10,000× *g* for 1 h at 4 °C, and the supernatant was discarded, and the pellet was resuspended in PBS or ultrapure water (for PKH26 labeling). The isolated exosomes were kept at 4 °C for up to 1 week.

### 4.5. Library Preparation and Sequencing

Total RNA was isolated using TRIzol reagent (Invitrogen, Carlsbad, CA, USA). Total exosomes containing RNAs were isolated using LS Trizol reagent (Invitrogen, Carlsbad, CA, USA). RNA quality was assessed using an Agilent 2100 Bioanalyzer using an RNA 6000 Nano Chip (Agilent Technologies, Santa Clara, CA, USA), and RNA quantification was performed spectrophotometrically (ND-2000; Thermo Fisher Scientific, Waltham, MA, USA). Libraries were prepared from 2 µg of total RNA using the SMARTer Stranded RNA-Seq Kit (Takara Bio, Shiga, Japan) [51]. mRNAs were isolated using the Poly(A) RNA Selection Kit (LEXOGEN, Inc., Vienna, Austria). The isolated mRNAs were used for cDNA synthesis and shearing following the manufacturer’s instructions. A small RNA-seq cDNA library was constructed using the NEB Next Multiplex Small RNA Library Prep kit (New England Biolabs, Ipswich, MA, USA), and the average Q20 was calculated using BBDuk (version 38.90, Joint Genome Institute, Berkeley, CA, USA). Indexing was performed using the Illumina (Illumina, Inc., San Diego, CA, USA) indices 1–12. Enrichment was performed by PCR. Subsequently, libraries were checked using the Agilent 2100 Bioanalyzer (DNA High Sensitivity Kit; Agilent Technologies, Santa Clara, CA, USA) to evaluate the mean fragment size. Quantification was performed using a library quantification kit and the StepOne Real-Time PCR System (Life Technologies, Carlsbad, CA, USA). High-throughput sequencing was performed as paired-end 100 sequencing using HiSeq 2500 (Illumina, San Diego, CA, USA).

### 4.6. GFP Labeling of Dgcr8 KO mESCs

To generate GFP-labeled Dgcr8 knockout (KO) mESCs, cells were transduced with a GFP-expressing lentiviral vector to establish a stable GFP-marked cell population. The lentiviral GFP plasmid was propagated in Stbl3 competent cells to ensure stable maintenance of lentiviral plasmids containing LTR elements. Lentiviral particles were produced and used to transduce Dgcr8 KO mESCs. Following transduction, GFP-positive cells were isolated by fluorescence-activated cell sorting (FACS) and expanded for subsequent experiments.

### 4.7. Flow Cytometry Analysis

We used flow cytometry (FACS Canto II, BD Biosciences, Franklin Lakes, NJ, USA) to detect the indicated fluorochromes. Positive and negative controls were obtained from the TUNEL assay kit (Invitrogen, Waltham, MA, USA), and 7-AAD stained samples were collected in a FACS tube (Corning Life Sciences, Durham, NC, USA). The fluorescence intensities of bromodeoxyuridine (BrdU)-Red (excitation/emission = 488/576 nm) and 7-AAD (excitation/emission = 488/655 nm) were detected and used to determine the percentage difference between each experimental group.

### 4.8. Terminal Deoxynucleotidyl Transferase-Mediated Deoxyuridine Tri-Phosphate (dUTP) Nick End Labeling (TUNEL) Assay

EBs of v6.5 and *Dgcr8* KO mESCs and exosome-treated *Dgcr8* KO mESCs were dissociated by trypsinization. All dissociated cells were washed twice with PBS. TUNEL staining was performed using an APO-BrdU TUNEL Assay Kit (Invitrogen, Waltham, MA, USA) following the manufacturer’s instructions. The stained cells were detected by flow cytometry.

### 4.9. Genomic DNA Extraction and Genotype PCR Analysis

mESCs were lysed (Tris-HCl 1 M, EDTA 0.5 M, SDS 20%, NaCl 5 M, and ddH_2_O) using proteinase K and RNase A for 10 min and incubated at 60 °C. Genomic DNA was extracted from mESC pellets using an AccuPrep Genomic DNA Extraction Kit (BIONEER, Daejeon, Republic of Korea). Each PCR reaction was performed using 50 ng of genomic DNA, and the PCR products were separated on 1% agarose gel containing ethidium bromide [25].

### 4.10. PKH26 Labeling

Lyophilized exosome standards were purified from cell culture media. For PKH26 labeling, exosomes were resuspended in ultra-pure water to a protein concentration of 40 μg/mL. The resuspended exosomes were stained with PKH26 using the PKH26 Red Fluorescent Cell Linker Kit for general cell membrane labeling (Sigma, St. Louis, MO, USA). The volume of purified exosomes was increased to 1 mL using Diluent C for each sample. Thereafter, 6 μL PKH26 dye was added to each of the 1 mL Diluent C tubes, followed by mixing for 30 s by gentle pipetting and incubating at room temperature for 5 min. Next, 1.5 mL of the 0.971 M sucrose solution was added by carefully pipetting into the bottom of the tube (exosomes and PKH26 solution remained on top of the sucrose) and then centrifuged at 190,000× *g* for 2 h at 4 °C. The red pellet remained at the bottom of the tube, and the supernatant and interface layer were carefully aspirated. The exosome pellet was resuspended in PBS by gentle pipetting, followed by transfer to an Amicon 10 kDa MWCO (Millipore^TM^, Burlington, MA, USA) filter column and centrifugation at 3000× *g* for 40 min. To recover concentrated exosomes, the Amicon Ultra filter device (MilliporeSigma, Burlington, MA, USA) was placed upside down in a new tube, and then centrifugation was performed for 2 min at 1000× *g* to transfer the concentrated exosomes from the device to the tube.

### 4.11. Cell Cycle Assay

Cell cycle analysis was performed using 7-AAD staining. The harvested cells were washed in PBS and fixed in ice-cold 70% pure ethanol and incubated at 4 °C overnight. Cells were treated with ribonuclease A at 100 μg/mL (R6148; QIAGEN, Hilden, Germany), pelleted at approximately 2000 rpm for 5 min, and washed twice in PBS. Thereafter, 7-AAD viability staining solution (cat. 00-6993) was added under protection from light. The fluorescence intensity of 7-AAD was visualized using the cell cycle tool in the FlowJo software (version 10.7.1, BD Biosciences, Ashland, OR, USA) and was used to determine the percentage difference between each experimental group.

### 4.12. GSEA

Candidate miRNA target mRNAs, regardless of whether they were differentially expressed in each group, were used for GSEA. Gene set analysis was performed using the GSEA software (https://www.gsea-msigdb.org/gsea/index.jsp (accessed on 24 August 2021); version 4.1.0, Broad Institute, Cambridge, MA, USA) and MSigDB v7.4. A normalized enrichment score (NES) was determined for each gene set [52]. Significant enrichment of a gene set was based on absolute value of NES > 1, nominal *p*-value of NES ≤ 0.05, and false discovery rate ≤ 0.05.

### 4.13. Statistical Analysis

All experiments were independently repeated at least three times. Statistical analyses were performed using GraphPad Prism 8.0 software (GraphPad, Inc., San Diego, CA, USA). Data are presented as means ± standard deviation (SD). For comparisons involving more than two groups, statistical differences were analyzed by two-way or one-way analysis of variance with Tukey’s post hoc test; *p* < 0.05 defined statistical significance.

## 5. Conclusions

Pluripotent stem cells represent a powerful platform to investigate early development and advance regenerative medicine. A clear understanding of the mechanisms governing stem cell differentiation is essential for harnessing their full potential. This study identified exosomal miRNAs as key regulators of early embryonic differentiation. Using *Dgcr8* KO ESCs, which lack canonical miRNAs, we demonstrated that exosomes derived from differentiating ESCs could restore trilineage differentiation, morphology, and apoptosis control. These findings suggested that exosomal miRNAs act as paracrine morphogen-like signals that guide cell fate transitions. Our results support a novel framework in which extracellular vesicles, specifically their miRNA cargo, can compensate for intrinsic deficits and coordinate differentiation. This study provides new insights into developmental biology and opens new avenues for miRNA-based modulation in stem cell engineering and therapy.

## Figures and Tables

**Table 1:** Dgcr8 exon 3 target primer sequences.

Primer Target	Sequence Information
Dgcr8p3.1	CTGGAGTAGGAATGTTGATTTC
Dgcr8loxr	CCTGATTCACTTACAACACAACC
Dgcr8wr	TAAAGCGTCCACATCATTGTC

## Data Availability

The datasets generated and analyzed in the current study are available from the corresponding author upon reasonable request.
